# Effects of prolonged drought on the anatomy of sun and shade needles in young Norway spruce trees

**DOI:** 10.1002/ece3.1766

**Published:** 2015-10-15

**Authors:** Roman Gebauer, Daniel Volařík, Josef Urban, Isabella Børja, Nina Elisabeth Nagy, Toril Drabløs Eldhuset, Paal Krokene

**Affiliations:** ^1^ Departement of Forest Botany, Dendrology and GeobiocoenologyMendel University in BrnoZemědělská 361300BrnoCzech Republic; ^2^ Norwegian Institute of Bioeconomy ResearchPO Box 115N‐1431ÅsNorway

**Keywords:** Climatic change, drought, hydraulic conductivity, light quality, needle growth, *Picea abies*, tracheid, xylem transport

## Abstract

Predicted increases in the frequency and duration of drought are expected to negatively affect tree vitality, but we know little about how water shortage will influence needle anatomy and thereby the trees’ photosynthetic and hydraulic capacity. In this study, we evaluated anatomical changes in sun and shade needles of 20‐year‐old Norway spruce trees exposed to artificial drought stress. Canopy position was found to be important for needle structure, as sun needles had significantly higher values than shade needles for all anatomical traits (i.e., cross‐sectional needle area, number of tracheids in needle, needle hydraulic conductivity, and tracheid lumen area), except proportion of xylem area per cross‐sectional needle area. In sun needles, drought reduced all trait values by 10–40%, whereas in shade needles, only tracheid maximum diameter was reduced by drought. Due to the relatively weaker response of shade needles than sun needles in drought‐stressed trees, the difference between the two needle types was reduced by 25% in the drought‐stressed trees compared to the control trees. The observed changes in needle anatomy provide new understanding of how Norway spruce adapts to drought stress and may improve predictions of how forests will respond to global climate change.

## Introduction

In central and eastern Europe, climate change is predicted to increase the frequency and duration of summer droughts (EEA 2015), highlighting the need for more knowledge about how trees respond to drought. Norway spruce (*Picea abies* (L.) Karst.) is the dominant forest tree over much of Europe and the most important commercial tree species in many countries. At the same time, Norway spruce is not particularly drought tolerant and is thus one of the tree species that may be most vulnerable to climate change. Indeed, dieback of Norway spruce is already observed many places in Europe (Hentschel et al. 2014), and drought stress is suggested to be the driving factor behind this process (Allen et al. 2010).

The ability of trees to survive drought depends on a variety of phenological, morphological, and physiological factors. These factors induce multiple short‐ and long‐term responses, including stomatal closure (Lu et al. 1996), changed expression of dehydrin genes (Eldhuset et al. 2013), inhibition of shoot growth (Chaves et al. 2003), and reduced root longevity (Meier and Leuschner 2008). Important longer‐term adaptations to drought are anatomical responses in water transporting tissues, such as a reduction in tracheid lumen area (Abe and Nakai 1999), that modify hydraulic structure in ways that may reduce the risk of hydraulic failure. There have been many studies of drought responses of Norway spruce focusing on gas exchange (e.g., Dixon et al. 1998), sap flow (e.g., Gartner et al. 2009), biomass accumulation (e.g., Solberg 2004), growth (e.g., Jyske et al. 2010), terpenoids (e.g., Turtola et al. 2003), hydraulic conductance (e.g., Lu et al. 1996), nutrient uptake (e.g., Nilsson and Wiklund 1994), ectomycorrhizae (e.g., Nilsen et al. 1998), or cell organelles (e.g., Kivimäenpää et al. 2003). However, studies on anatomical adaptation of Norway spruce needles to drought are rare and seem to be limited to our recent greenhouse study on 5‐year‐old plants (Eldhuset et al. 2013) and a study on needle vascular cylinder area in young saplings in open‐top chambers (Kivimäenpää et al. 2003).

In addition to drought, light availability is an important factor modifying needle development. The plastic responses of conifer needles to different irradiance levels have been thoroughly studied, and compared to sun needles, shaded needles have lower density (Niinemets and Kull 1995), width (Gebauer et al. 2011), or mesophyll thickness (Youngblood and Ferguson 2003). However, even though light and water availability are the two main determinants of needle anatomy, no one has investigated whether there are any interacting effects of these two parameters on cross‐sectional needle area, needle phloem and xylem area, number of tracheids in needle, needle hydraulic conductivity, tracheid lumen area, or tracheid diameter.

**Figure 1:**
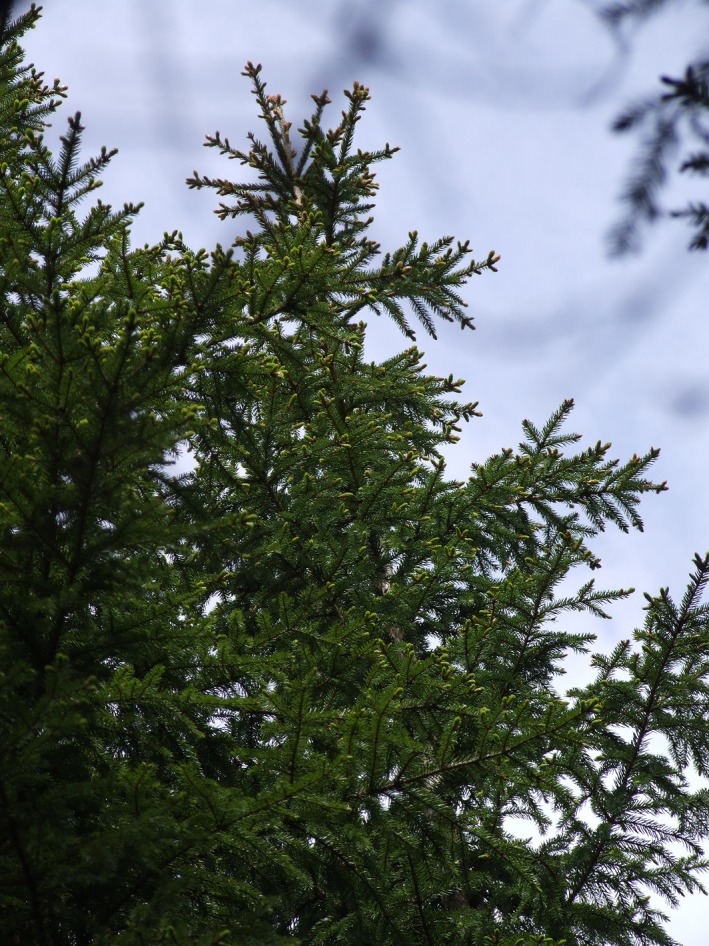
Young (~20 years old) Norway spruce trees from the experimental plot in Ås, Norway.

This study aimed to characterize the effects of prolonged drought on the anatomical structure of sun and shade needles in young (~20 years old) Norway spruce trees (Fig. 1). Increased knowledge about the underlying mechanisms of needle development in response to drought is crucial for understanding and modeling adaptation mechanisms in Norway spruce. We tested the following three hypotheses: (1) drought will change needle anatomical traits in both sun and shade needles as a result of water shortage during cell elongation; (2) sun needles will be more affected by drought than shade needles, because of their higher water demand, and (3) this stronger response in sun needles will result in smaller differences between sun and shade needles in trees subjected to drought than in control trees.

## Materials and Methods

### Study site and experimental design

This study follows up previous studies of anatomical changes in Norway spruce shoots and needles following thinning (Gebauer et al. 2011, 2012, 2014). We sampled needles from the same trees used in the previous studies, and further details about the study site and experimental design can be found in Gebauer et al. (2011 and 2012). Briefly, we used ~20‐year‐old Norway spruce trees that had been planted in a 1 × 1 m grid on former agricultural soil in 1990 in Ås, SE Norway. Mean stand height was 9 m, stand density was 10,000 trees ha^−1^, and basal area was about 50 m^2^ ha^−1^. Two study plots, about 150 m^2^ each and situated ~15 m apart, were established within the stand. In each plot, we selected one tree from each of the same three full‐sib families (families 15, 27, and 29; see Gebauer et al. 2011 for details). Trees standing along the edges of the plots were not selected. One plot received natural rainfall and served as the control plot. On the other plot (drought plot), the three selected trees were standing in one row and the two tree rows flanking the experimental trees were removed in August 2008. In May 2009, a plastic roof covering the entire drought plot was set up under the tree crowns and a 30‐cm‐deep trench was dug around the plot to intercept the precipitation. The trench was dug outside the flanking tree rows to avoid root damage on the experimental trees.

Sap flow was monitored from mid‐May to the end of August, 2010, in all six experimental trees, using the EMS 51 sap flow meters (EMS Brno, Czech Republic) working under the trunk heat balance principle (Čermák et al. 1973; Kučera et al. 1977). A more detailed description of measurement of sap flow, tree, and soil characteristics is given in Gebauer et al. (2011). We monitored SWP (soil water potential) in triplicate measurements on both plots at 10, 30, and 50 cm soil depth using gypsum blocs (Delmhorst Inc., Towaco, NJ). Data were acquired every 10 min and stored in a data logger (Modulog 1037, EMS Brno, Czech Republic). SWP was measured from the beginning of the vegetation period until all samples were taken (i.e., from May to August 2010). A climatic station was set up on an open field of 100 m from the experimental plots to measure global radiation, air temperature, and relative air humidity using EMS11 and EMS33 (EMS Brno, Czech Republic), as well as precipitation and wind speed (MetOne Instruments, Grants Pass, OR). Data were measured every minute, and 10‐min means were stored in a data logger. Vapor pressure deficit and Penman‐Monteith‐based reference evapotranspiration were calculated from the weather data as described by Allen et al. (1998).

### Light intensity

The amount of PAR (photosynthetically active radiation) in the two stands was measured under cloudless conditions on 4 August 2010. PAR measurements were taken twice (i.e., around noon and in the late afternoon) to assess radiation interception at different elevations of the sun. Measurements were taken with two logging PAR sensors (Minikin QT, EMS Brno, Czech Republic): One sensor was left in the open area outside the forest to measure ambient solar radiation, and the other sensor was held by a person walking slowly through the plot. The measurement interval was set to 3 sec, and mean PAR values were calculated. Plots were compared using ratios [%] of plot PAR to ambient PAR.

### Plant material

From each tree, five current‐year shoots were collected near the top of the tree (sun needles; upper 1–2 m of the crown) and five current‐year shoots were collected at the bottom of the crown (shade needles; ~4 m above ground) on 7 August 2010, that is, 15 months after roof installation. Shoots were always sampled from the outer part of the southern side of the crown to minimize the effect of any microclimatic differences within the stand or canopy (Lhotáková et al. 2007). Samples were fixed in FAA solution (90 mL of 70% ethanol, 5 mL of acetic acid, and 5 mL of 40% formaldehyde).

### Needle analysis

**Figure 2:**
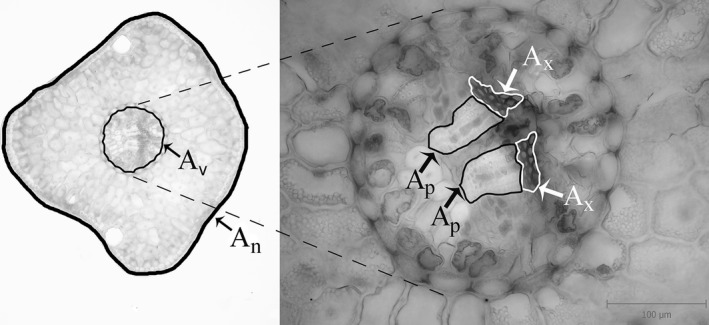
Micrograph of a sun needle from Norway spruce family 14 showing some of the measured needle traits: cross‐sectional needle area (*A*_n_), vascular cylinder area (*A*_v_), phloem area (*A*_p_), and xylem area (*A*_x_).

Seven needles per shoot were taken for detailed anatomical measurements. Needles were collected at regular intervals along the shoot axis to cover possible within‐shoot heterogeneity in needle morphology. All together, 35 needles from each tree height were analyzed (seven needles × five shoots). Needle cross sections for anatomical analyses were taken near the needle base and dyed using a saturated solution of phloroglucinol in 20% hydrochloric acid (HCl) that highlights lignified cell walls in red. Stained sections were examined under a light microscope (Olympus BX51, Olympus Czech Group, Corporation, Prague, Czech Republic) and photographed using a digital camera (Olympus E‐330, Olympus Czech Group, Corporation) connected to a computer by QuickPhotomicro 2.3 software (Promicra, Czech Republic). Cross‐sectional needle area (*A*_n_), vascular cylinder area (*A*_v_), xylem area (*A*_x_), and phloem area (*A*_p_) (Fig. 2) were determined from the sections using ImageJ 1.45 analyzing software (The University of Texas Health Science Center, San Antonio, TX). Tracheid lumina were manually colored using Adobe Photoshop 9.0 (Adobe Systems, Inc, San Jose, CA), and tracheid lumen area (*A*_lum_) and maximum (*d*_max_) and minimum (*d*_min_) tracheid lumen diameter were measured using ImageJ. Tracheid flatness (*F*_t_) was calculated as the ratio *d*_max_/*d*_min_. We also quantified the number of tracheids per needle cross‐section (*N*_t_), total tracheid lumen area per needle cross section (*N*_lum_), and proportion of xylem and phloem per cross‐sectional needle area (*A*_x_^%^ and *A*_p_^%^, respectively).

The theoretical hydraulic conductivity of each tracheid (*k*_t_) was calculated according to the Hagen–Poiseuille law (equation (1)). Because the cross section of the tracheid lumens was elliptical, a modification to the formula was applied as recommended by Martre et al. (2000) (equation (2)).

(1) $$k_{t}=\left(\pi \rho /8\eta \right)\;r_{lum}^{4}\;\left[\text{kg}\;m\;{\text{sec}}^{-1}\;{\text{MPa}}^{-1}\right],$$

(2) $$r_{lum}^{4}=d_{max}^{3}d_{\min}^{3}/\left({8d}_{\max}^{2}+\;{8d}_{\min}^{2}\right),$$

where *ρ* is the density of water at 20°C (998.205 kg m^−3^), *η* is the viscosity of water at 20°C (1.002 × 10^−9^ MPa sec), and *r*_*lum*_ is the lumen radius.

The theoretical hydraulic conductivity of all tracheids per needle cross section (*k*_th_) was calculated as the sum of all *k*_t_ per needle. Needle xylem‐specific hydraulic conductivity (*k*_s_) was then calculated as *k*_th_ divided by *A*_x_. All abbreviations used in this study are shown in Table 1.

**Table 1:** The anatomical and hydraulic traits of Norway spruce needles quantified in this study

Variable	Explanation	Unit
*A*_lum_	Tracheid lumen area	*μ*m^2^
*A*_n_	Cross‐sectional needle area	mm^2^
*A*_p_	Phloem area	*μ*m^2^
*A*_p_^%^	Proportion of phloem area per cross‐sectional needle area	%
*A*_v_	Vascular cylinder area	mm^2^
*A*_x_	Xylem area	*μ*m^2^
*A*_x_^%^	Proportion of xylem area per cross‐sectional needle area	%
*d*_max_	Maximum tracheid lumen diameter	*μ*m
*d*_min_	Minimum tracheid lumen diameter	*μ*m
*F*_t_	Tracheid flatness (*d*_max_/*d*_min_)	–
*k*_t_	Tracheid theoretical hydraulic conductivity (Eq. (1))	kg m sec^−1^ MPa^−1^
*k*_th_	Needle theoretical hydraulic conductivity	kg m sec^−1^ MPa^−1^
*k*_s_	Needle xylem‐specific hydraulic conductivity	kg m^−1^ sec^−1^ MPa^−1^
*N*_lum_	Total lumen area per needle cross‐section	*μ*m^2^
*N*_t_	Number of tracheids in needle	–

### Statistical analysis

Differences in accumulated sap flow between control trees and trees subjected to drought were analyzed using a two‐sample two‐tailed *t*‐test at the 0.05 significance level. For the analysis of needle anatomical traits, we used LME (linear mixed effect) models (Zuur et al. 2009). LME was used because of our hierarchical sampling strategy, where needles were collected from different trees and from five different shoots on each tree. LME allows us to include variation among and within trees and shoots by specifying individual shoots within trees as a random effect. Traits measured at the tracheid level were averaged (e.g., *d*_max_) or summed (e.g., *k*_th_) to obtain average or total values per needle. Data on *k*_th_ were log‐transformed before analysis to fulfill the assumptions of LME. The explanatory variables needle type (sun/shade) and drought treatment were set as fixed effects. To test for the significance of these fixed effects and obtain appropriate *P*‐values, we used the approach described by Zuur et al. (2009). Briefly, likelihood ratio tests were used to compare each simpler model with a relevant more complex model. Statistical analyses were carried out in the R software environment (R Core Team 2015), using the package “lme4” version 1.1‐7 (Bates et al. 2014) for LME calculations and the package “lmerTest” version 2.0‐25 (Kuznetsova et al. 2015) for calculation of 95% confidence intervals of population means for fixed effects.

## Results

### Meteorology

**Figure 3:**
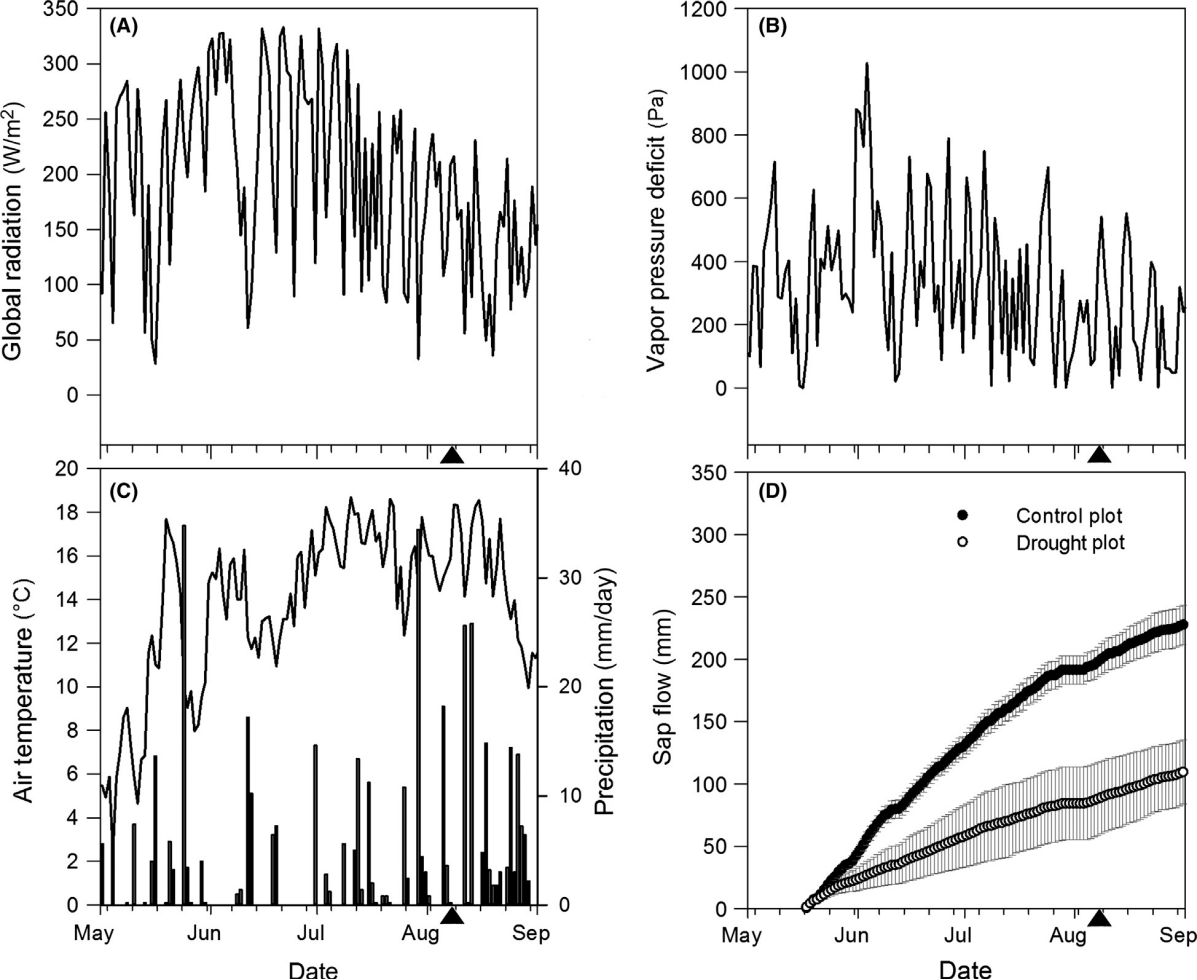
Meteorological conditions and sap flow in our experimental plots in 2010: daily means of global radiation (A), vapor pressure deficit (B), air temperature and precipitation (C), and cumulative sap flow in Norway spruce trees subjected to drought (white dots) and control trees (black dots) (±standard error of daily mean values) (D). Black arrows show the date needles were sampled for anatomical analyses.

The 2010 growing season, when the needles used in this study developed, was characterized by even rainfall without any long periods of drought (Fig. 3A–C). Mean air temperature from 1 May to 7 August (the day of needle sampling) was 13.6°C, mean vapor pressure deficit 347 Pa, total precipitation 270 mm, and Penman‐Monteith‐based reference evapotranspiration 332 mm.

### Soil water potential, sap flow, and PAR

Water availability in the soil differed strongly between the plots: Soil water was unlimited throughout most of the season in the control plot, whereas it was extremely low in the drought plot. Between May and August, SWP in the control plot averaged −0.1, −0.08, and −0.02 MPa at 10, 30, and 50 cm soil depth, respectively, and with the exception of 16 days in July, it never dropped below −0.02 MPa at any soil depth. In contrast, SWP in the drought plot was always below the wilting point (i.e., −1.5 MPa) at 10 and 30 cm soil depth. However, some water remained available at 50 cm soil depth, where SWP averaged −0.41 ± 0.06 MPa from May to August.

Mean accumulated sap flow per unit crown projected area was higher in the control plot than that in the drought plot (*P* < 0.001). Sap flow totaled 198 mm in the control plot and 87 mm in the drought plot from 17 May to 7 August (Fig. 3D). Thus, tree sap flow per unit crown projected area was reduced by 56% due to drought. The proportion of PAR reaching the bottom of the canopy varied between 16% of the PAR in the open area around noon and 2% in the late afternoon.

### Effects of drought on needle anatomy

Drought significantly reduced tracheid lumen diameter (*d*_max_) of both sun and shade needles by 10 and 5%, respectively, and significantly increased the proportion phloem per needle area (*A*_p_^%^) of both needle types by 18 and 6%, respectively (Table 2). For all other anatomical traits, sun and shade needles showed different responses to drought, and the interaction between drought and needle type was significant for nine of the 14 studied needle traits (Table 3). Vascular cylinder area (*A*_v_), phloem area (*A*_p_), proportion of xylem area per cross‐sectional needle area (*A*_x_^%^), tracheid flatness (*F*_t_), and number of tracheid in needle (*N*_t_) were the only variables that were not influenced by drought. In‐depth analysis of the drought × needle type interaction showed that sun needles generally were more affected by drought than shade needles (Table 2). The largest influence of drought on sun needles was observed for needle theoretical hydraulic conductivity (*k*_th_), needle xylem‐specific hydraulic conductivity (*k*_s_), and total lumen area per needle cross section (*N*_lum_), which were reduced by 41%, 32%, and 27%, respectively, in trees subjected to drought relative to control trees (Table 2). Drought also reduced cross‐sectional needle area (*A*_n_), xylem area (*A*_x_), and tracheid lumen area (*A*_lum_) of sun needles by ~20%, and minimum tracheid lumen diameter (*d*_min_) by 10% (Table 2).

**Table 2:** Anatomical and hydraulic traits of sun and shade needles from Norway spruce trees subjected to drought or control trees receiving natural rainfall. See Table 1 for an explanation of needle traits

Needle trait	Shade needles		Sun needles	
	Mean (95% confidence interval)		Mean (95% confidence interval)	
	Control	Drought	Control	Drought
*A*_lum_	14.5 (9.6–19.5)	13.1 (8.2–18.0)	20.2 (15.2–25.1)	16.3 (11.3–21.2)
*A*_n_	0.35 (0.29–0.42)	0.34 (0.28–0.40)	0.76 (0.69–0.82)	0.63 (0.57–0.69)
*A*_p_ (×10^2^)	12.7 (7.4–18.0)	13.1 (7.8–18.4)	29.4 (24.0–34.5)	28.7 (23.4–34.1)
*A*_p_^%^	0.37 (0.27–0.47)	0.39 (0.30–0.50)	0.39 (0.29–0.49)	0.47 (0.36–0.56)
*A*_v_ (×10^−3^)	19 (14–24)	17 (12–22)	48 (43–53)	42 (37–47)
*A*_x_ (×10^2^)	9.3 (6.5–12.1)	9.0 (6.2–11.7)	20.6 (17.8–23.4)	17.0 (14.2–19.7)
*A*_x_^%^	0.27 (0.22–0.33)	0.27 (0.21–0.32)	0.27 (0.22–0.33)	0.27 (0.21–0.32)
*d*_max_	5.2 (4.4–6.0)	4.9 (4.1–5.7)	6.2 (5.4–7.0)	5.6 (4.8–6.4)
*d*_min_	3.4 (2.9–3.9)	3.2 (2.7–3.7)	3.8 (3.3–4.3)	3.5 (3.0–3.9)
*F*_t_	1.61 (1.55–1.66)	1.60 (1.55–1.66)	1.75 (1.69–1.81)	1.72 (1.67–1.77)
*k*_th_ (×10^−11^)	18 (10–32)	14 (8–26)	61 (34–109)	36 (20–64)
*k*_s_ (×10^−12^)	0.22 (0.10–0.33)	0.18 (0.06–0.30)	0.34 (0.23–0.46)	0.23 (0.11–0.35)
*N*_lum_	291 (145–437)	263 (118–409)	713 (567–860)	519 (373–665)
*N*_t_	20.4 (18.2–22.6)	20.0 (17.9–22.1)	35.1 (32.8–37.5)	31.9 (29.7–34.1)

**Table 3:** Summary of linear mixed effect analyses for needle traits in sun and shade needles from Norway spruce trees subjected to drought or control trees receiving natural rainfall. Effects that are significant at the 5% level are highlighted in boldface. See Table 1 for an explanation of the different needle traits

Needle trait	Factors	AIC^a^	*P*‐value
*A*_lum_	**canopy height**	**1800**	**<0.001**
	drought	1849	0.08
	**drought × canopy height**	**1793**	**0.003**
*A*_n_	**canopy height**	**9496**	**<0.001**
	drought	9570	0.27
	**drought × canopy height**	**9492**	**0.02**
*A*_p_	**canopy height**	**5774**	**<0.001**
	drought	5874	0.97
	drought × canopy height	5778	0.89
*A*_p_^%^	**canopy height**	−**800**	**0.01**
	**drought**	−**798**	**0.04**
	**drought × canopy height**	−**803**	**0.03**
*A*_v_	**canopy height**	**7695**	**<0.001**
	drought	7773	0.41
	drought × canopy height	7692	0.21
*A*_x_	**canopy height**	**5382**	**<0.001**
	drought	5447	0.26
	**drought × canopy height**	**5379**	**0.03**
*A*_x_^%^	canopy height	−1162	0.93
	drought	−1162	0.9
	drought × canopy height	−1158	0.97
*d*_max_	**canopy height**	**515**	**<0.001**
	**drought**	**576**	**0.04**
	**drought × canopy height**	**509**	**0.007**
*d*_min_	**canopy height**	**110**	**<0.001**
	drought	152	0.09
	**drought × canopy height**	**105**	**0.016**
*F*_t_	**canopy height**	−**469**	**<0.001**
	drought	−433	0.54
	drought × canopy height	−466	0.57
*k*_th_	**canopy height**	**352**	**<0.001**
	drought	419	0.06
	**drought × canopy height**	**347**	**0.01**
*k*_s_	**canopy height**	−**22,229**	**<0.001**
	drought	−22,198	0.09
	**drought × canopy height**	−**22,237**	**0.003**
*N*_lum_	**canopy height**	**4652**	**<0.001**
	drought	4708	0.09
	**drought × canopy height**	**4645**	**0.004**
*N*_t_	**canopy height**	**2267**	**<0.001**
	drought	2333	0.51
	drought × canopy height	2267	0.15

a Akaike's information criterion.

### Effects of canopy height on needle anatomy

There were significant differences between sun and shade needles for all studied traits except proportion xylem per needle area (*A*_x_^%^) (Table 2). In general, needle traits were more extensive in sun needles than in shade needles. For cross‐sectional needle area (*A*_n_) and xylem area (*A*_x_), the larger dimension of sun needles was more pronounced in control trees (~2.2‐fold) than in trees subjected to drought (~1.8‐fold) (Table 2). Sun needles also had ~2.5‐fold and ~2.3‐fold larger vascular cylinder (*A*_v_) and phloem area (*A*_p_), respectively, than shade needles on both plots (Table 2). The proportion xylem per needle area (*A*_x_^%^) was only 0.27% across both needle types and drought treatments (Table 2). Proportional phloem area (*A*_p_^%^) was ~1.5 times larger than *A*_x_^%^, and sun needles had somewhat higher *A*_p_^%^ than shade needles, both in the control trees (3%) and in the trees subjected to drought (13%) (Table 2).

Sun needles had 1.7 times more tracheids than shade needles across both drought treatments (*N*_t_; Table 2). Sun needles also had larger tracheid lumen diameters (*d*_max_, *d*_min_) and tracheid lumen area (*A*_lum_), particularly in control trees (Table 2). Due to their more numerous and larger tracheids, total lumen area per needle cross section (*N*_lum_) was also significantly larger in sun needles than in shade needles, particularly in control trees (Table 2). Tracheid cross sections were ellipsoidal in both needle types, with tracheid flatness (*F*_t_) values from 1.60 to 1.75, and sun needles were somewhat flatter tracheids than shade needles (Table 2). The higher maximum tracheid lumen diameter (*d*_max_) and tracheid number (*N*_t_) in sun than in shade needles resulted in much higher theoretical hydraulic conductivity (*k*_th_) for sun needles, both in control trees (~3.4‐fold higher) and in trees subjected to drought (~2.5‐fold higher) (Table 2). Needle xylem‐specific hydraulic conductivity (*k*_s_) differed much less between sun and shade needles than *k*_th_ (Table 2), because xylem area (*A*_x_) was higher in sun needles than in shade needles. In general, the anatomical differences between sun and shade needles were reduced by 25% in trees subjected to drought compared to control trees.

## Discussion

### Effects of drought on the anatomical structure of shade needles

Drought stress during needle development typically leads to reduced cell dimensions due to loss of turgor in the cell elongation phase (Chaves et al. 2003). In our study, however, reduced cell dimensions were only observed in sun needles, whereas shade needles showed very modest anatomical responses to drought. Shade needles had slightly lower (~5%) maximum tracheid lumen diameter (*d*_max_) in trees subjected to drought than in control trees (Table 2). A decrease in *d*_max_ is usually connected to an increase in hydraulic resistance and could be an adaption to reduce water loss in the shade needles (Tyree and Zimmermann 2002). However, in our study, a slightly lower *d*_max_ in shade needles had little effect on the theoretical hydraulic conductivity (*k*_th_), as the tracheid number (*N*_t_) was very similar in drought and control trees. The explanation for the weak response of shade needles to drought could be that shade needles already were physiologically constrained and operated close to their survival threshold (Sellin and Kupper 2004). As shade needles are small, with reduced photosynthesis and small tracheids, further reductions in anatomical dimensions might result in needle mortality, shedding, and eventual death of branches. If this was the case in our experimental trees, further drought stress would not have led to structural changes in shade needles, but rather to needle shedding. Some empirical studies suggest that shade needles operate close to their survival threshold and thus are highly susceptible to drought. Drought stress in closed canopy *Pinus contorta* trees led to increased mortality of foliage on shaded branches in the lower canopy relative to foliage in the upper canopy (Protz et al. 2000). Imposed drought also dramatically decreased biomass increment in seedlings of *Pinus canariensis* (Climent et al. 2006) and *Fagus sylvatica* (Löf et al. 2005) in shaded environments. Another explanation why shade needles did not adjust anatomically to drought conditions might be linked to optimization of whole‐tree photosynthesis under drought. Shade needles are adapted to maintain photosynthetic function under conditions of low light availability and low transpiration demand and thus experience less water stress than sun needles. By maintaining water transport capacity in shade needles under drought trees may therefore be able to maintain whole‐tree photosynthesis at a higher level than they would if shade needles were shed.

### Effects of drought on the anatomical structure of sun needles

Drought reduced most anatomical traits in sun needles by 10–45%, although tree sap flow per unit crown projected area was reduced even more, by 56%. The greater effect of drought on whole‐tree sap flow than on anatomical traits may be due to increased water‐use efficiency (Welander and Ottoson 2000), reduced shoot growth (Bréda et al. 2006), or needle shedding (Climent et al. 2006).

Mean tracheid lumen diameter (*d*_max_) in our sun needles was ~6 *μ*m. This is similar to the value found by Gebauer et al. (2012) (~5 *μ*m) and similar to the typical conduit diameter in conifer needles, as assessed by Hacke and Sperry (2001). Tracheid lumen diameter is a key trait determining hydraulic conductivity in trees, and variation in hydraulic conductivity of needles developing under different environmental conditions is determined by tracheid expansion and other growth‐related processes, such as cell division (Koch et al. 2004). In our case, drought did not influence tracheid cell division, as the number of tracheids (*N*_t_) was similar across drought treatments. Thus, reduced tracheid lumen diameters were the only reason for the lower theoretical hydraulic conductivity (*k*_th_) and lower efficiency of water transport through the xylem (*k*_s_) of drought‐stressed sun needles.

Hydraulic conductivity calculated from conduit diameters typically overestimates measured conductivity considerably, as the anatomy and interconnections of tracheids are more complex than the simple series of parallel straight‐walled tubes assumed in the calculation of *k*_th_ (Tyree and Zimmermann 2002). Measured conductivity in conifers has usually been found to be between 30 and 50% of the theoretical conductivity (Sperry et al. 2006). In our study, measured conductivity would probably have been in the upper part of this range (close to 50%) because tracheid diameters were much smaller than 20 *μ*m. When tracheid diameters are below 20 *μ*m, pit resistance to water flow is negligible and lumen resistance is the major resistance to flow (Hacke et al. 2005).

### Effects of canopy height on needle structure

Sun needles in the upper crown of our experimental trees had a much larger phloem area (*A*_p_) than shade needles at the crown base and should therefore be better at transporting photosynthates (Niinemets et al. 2007). As sun needles face higher irradiances than shade needles, they also have greater evaporative demands that probably require a larger xylem volume (Niinemets et al. 2007). This fits well with our observation that sun needles had ~2.3‐fold larger xylem area (*A*_x_) than shade needles. The anatomical dimensions of shade needles are probably more restricted by low photosynthetic rates (Protz et al. 2000) than by tree height (Woodruff et al. 2008).

The larger tracheid lumen area (*A*_lum_) of sun needles compared to shade needles was mostly due to larger maximum tracheid diameters (*d*_max_), whereas minimum tracheid diameters (*d*_min_) were less plastic. Larger tracheid diameter and numbers (*N*_t_) in sun needles fit with the higher calculated hydraulic conductivity (*k*_th_) we found for sun needles. Our data support Sellin and Kupper's (2004) general conclusion that differences in needle conductivity between the upper and lower canopy are a result of differences in needle xylem anatomy. Although sun needles have the advantage of higher conductivity, they may also be more susceptible to cavitation due to their wider tracheids (Domec et al. 2009; Johnson et al. 2009), or to implosion of tracheid cell walls when water potentials drop (Woodruff et al. 2007).

## Conclusions

Contrary to our first hypothesis that prolonged drought will decrease anatomical traits in both sun and shade needles in young Norway spruce trees, we found that drought mostly affected sun needles. The only exceptions to this pattern were maximum tracheid lumen diameter (*d*_max_) and proportion phloem per needle area (*A*_p_^%^), which decreased (*d*_max_) or increased (*A*_p_^%^) in both needle types. Our second hypothesis was supported, as we generally found a stronger reduction in anatomical traits in sun needles than in shade needles. The stronger effect of drought on sun needles resulted in smaller differences between sun and shade needles in trees subjected to drought than in control trees, supporting our third hypothesis. Our results show that needles in different parts of the canopy react to drought in different ways: Sun needles adapted to drought by undergoing anatomical changes, whereas such adaptations were not observed for shade needles. At a more general level, our findings may improve predictions of how forests will respond to global climate change. Nevertheless, further replication on other Norway spruce genotypes growing in different soil and environmental conditions would be useful to confirm the generality of our work.

## Conflict of Interest

The authors have declared that they have no conflict of interest.
